# Identification and Study of Biomarkers from Novichok-Inhibited Butyrylcholinesterase in Human Plasma

**DOI:** 10.3390/molecules26133810

**Published:** 2021-06-22

**Authors:** Woo-Hyeon Jeong, Jin-Young Lee, Kyoung-Chan Lim, Hyun-Suk Kim

**Affiliations:** Agency for Defense Development (ADD), P.O. Box 35, Yuseong-gu, Daejeon 34186, Korea; marlintime@add.re.kr (J.-Y.L.); bps178@add.re.kr (K.-C.L.); alchemist@add.re.kr (H.-S.K.)

**Keywords:** nerve agent, Novichok, LC-MS/MS, chemical warfare agents

## Abstract

To identify biomarkers of ethyl (1-(diethylamino)ethylidene)phosphoramidofluoridate (A234)- or methyl (1-(diethylamino)ethylidene)phosphoramidofluoridate (A232)-inhibited butyrylcholinesterase (BChE), we investigated nonapeptide adducts containing the active site serine, which plays a key role in enzyme activity, using LC-MS/HRMS. Biomarkers were acquired as expected, and they exhibited a significant amount of fragment ions from the inhibiting agent itself, in contrast to the MS2 spectra of conventional nerve agents. These biomarkers had a higher abundance of [M+2H]^2+^ ions than [M+H]^+^ ions, making doubly charged ions more suitable for trace analysis.

## 1. Introduction

Novichok is a new type of organophosphorus nerve agent (OPNA) and is highly toxic because it rapidly inactivates cholinesterase (ChE) activity [1]. Ethyl (1-(diethylamino)ethylidene)phosphoramidofluoridate (A234) was alleged by the British government to have been used to poison Sergei and Yulia Skripal in Salisbury, England, on March 4, 2018 [2]. Its identity was then confirmed by the Organization for the Prohibition of Chemical Weapons (OPCW) [3]. After this poisoning attack, Novichok was newly included in the Chemical Weapons Convention (CWC) list in June 2020. [4] The intention to use of these warfare agents was further found in the poisoning case of Alexei Navalny in 2020. These agents were initially confirmed by Vil Mirzayanov, and are also known as chemical agents developed under the FOLIANT project of the USSR [5,6].

Despite the recent discovery and the legal restrictions on the acquisition of these compounds, a series of studies have been conducted on their toxicity and physical proper-ties [6,7], simulated toxicity in silico [8], degradation conditions, and corresponding pathways [9,10,11].

Although the toxicity of A234 has not been proven explicitly, we speculate that it has a similar poisoning mechanism to OPNAs due to related studies and their chemical similarity. Acetylcholinesterase (AChE, P22303) and butyrylcholinesterase (BChE, P06276) are serine hydrolases, in which the active center contains a serine residue [12,13]. AChE is primarily responsible for enabling regular nerve function by hydrolyzing the neurotransmitter acetylcholine (ACh), and the inhibition of AChE by OPNAs results in a subsequent buildup of ACh, which blocks cholinergic nerve impulses, leading to paralysis, suffocation, and death [14]. Novichok agents were reported to exert nerve toxicity by inhibiting BChE activity according to the clinical report on a victim [1], which is supported by a deposited PDB structure of the A232-inhibited AChE structure whose active site is covalently blocked with an OPNA structure (PDB ID 6NTM). Thus, it is suggested that these agents intoxicate patients in a similar way to their ancestral relatives.

However, to the best of our knowledge, no detailed investigation has been carried out to identify biomarkers for Novichok in blood samples. As a first step toward identifying biomarkers of Novichok, we developed an analytical method to identify biomarkers of A232 and A234 in human plasma by liquid chromatography-tandem mass spectrometry (LC-MS/MS).

## 2. Results

### 2.1. Biomarker Preparation and Detection by LC-MS/MS

Conventional nerve agents, such as sarin (GB), soman (GD), or VX react with 202Ser of human acetylcholinesterase [15] to block the degradation of acetylcholine, causing failure of the nervous system. Recently, a crystal structure of human acetylcholinesterase complexed with Novichok agent A232 (PDB ID 6NTM) showed that A232 reacts with the same residue as other nerve agents. Since butyrylcholinesterase shares many characteristics with acetylcholinesterase, such as amino acid sequence, active site structure, and substrate structure, it is considered a good potential bioscavenger against nerve agents [16,17], and many verification studies support its reactivity against agents [18,19,20,21,22,23].

**Figure 1:**
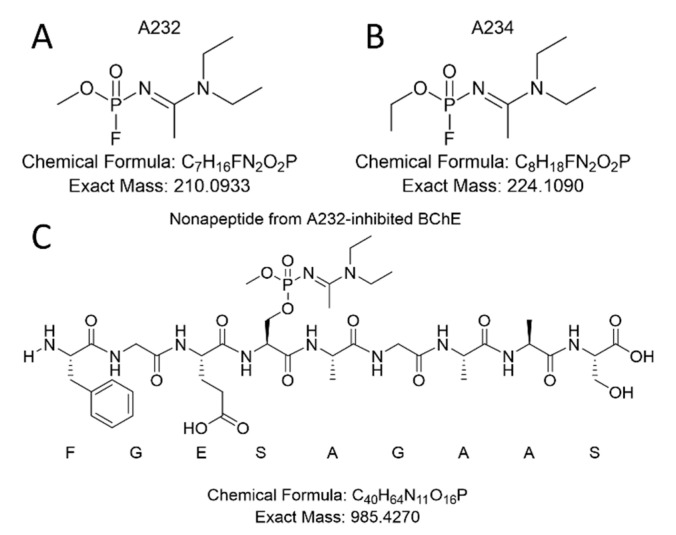
Chemical structure of A232 (**A**) and A234 (**B**), and the expected structure of the BChE biomarker (**C**).

**Figure 2:**
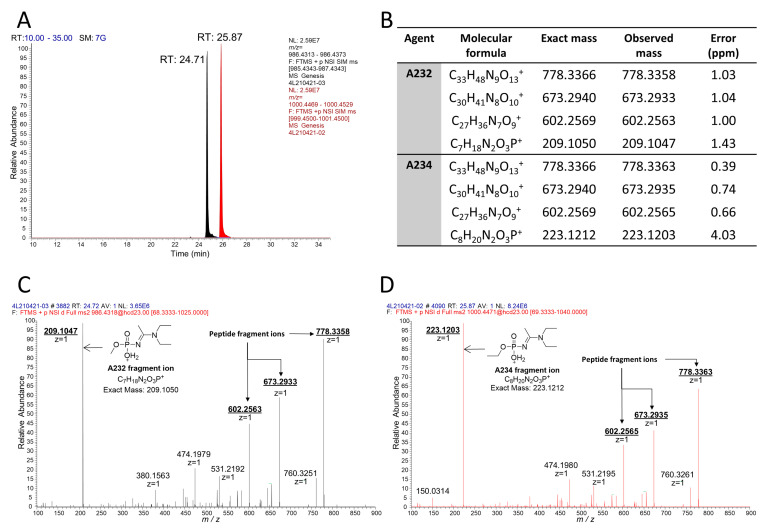
LC-MS/MS analysis of A232- and A234-inhibited BChE with pepsin digestion. (**A**) Extracted chromatograms from tSIM for biomarkers from A232 (black) and A234 (red). (**B**) Table of the major fragment ions in the MS2 spectra. MS2 spectra of the biomarkers from A232 (**C**) and A234 (**D**) show characteristic fragment ions from the nerve agent itself.

**Figure 3:**
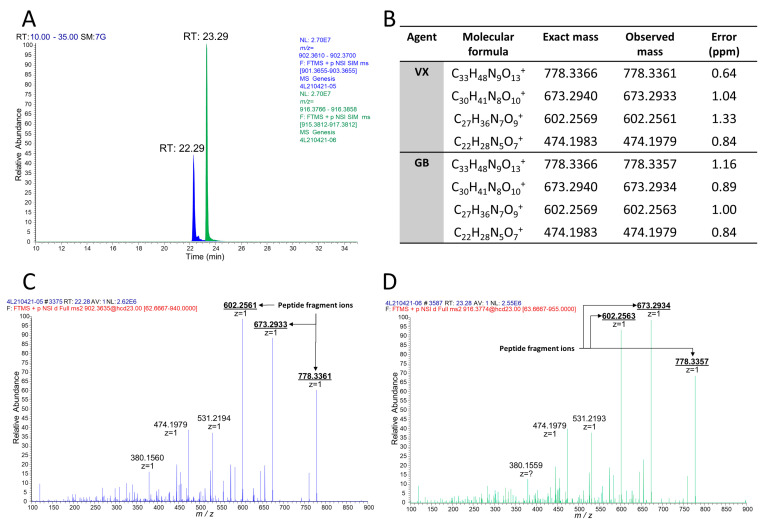
LC-MS/MS analysis of VX- or GB-inhibited BChE with pepsin digestion. (**A**) Extracted chromatograms from tSIM for biomarkers from VX (blue) and GB (green). (**B**) Table of major fragment ions in the MS2 spectra. MS2 spectra of the biomarkers from VX (**C**) and GB (**D**) are described below.

**Figure 4:**
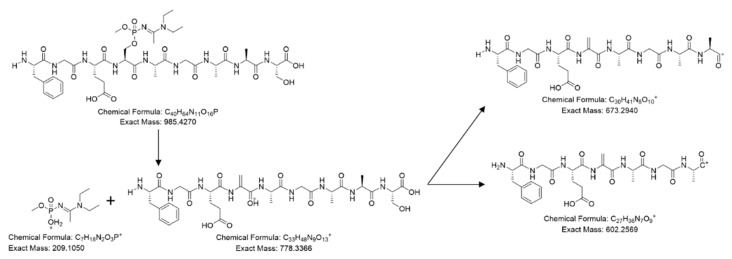
Fragmentation pathway of A232-inhibited nonapeptide from BChE by pepsin digestion.

From these studies, we postulated that Novichok agents inhibit the same active site of BChE as their conventional relatives do. Upon the assumption above, we predicted a possible nonapeptide structure inhibited with A232 or A234 based on their structure (Figure 1), calculated their exact mass, and conducted targeted selected ion monitoring (tSIM) analysis to determine whether the peptide truly exists. In the tSIM-data-dependent MS2 experiment, we found the predicted biomarker (Figure 2) and acquired its MS2 fragmentation pattern. The pattern supported that Novichok agents inhibit the same site of BChE as G- and V-series agents (Figure 3), and the major fragment ions are listed in the table (to be inserted). Interestingly, the MS2 pattern of the Novichok-inhibited biomarker exhibited a remarkable characteristic: it produced fragment ions from the inhibiting nerve agent itself. This characteristic can benefit the use of this biomarker for verification of the Novichok agent by providing clearer information. From the exact masses of the peptide fragment ions and the agent fragment ions, the C-O bond in the side chain of 198Ser, which is the active site of BChE, would fragmented first. The suggested fragmentation pathways based on the findings are described (Figure 4).

### 2.2. Identifying Multiply Charged Molecular Ions for MS/MS Analysis

**Figure 5:**
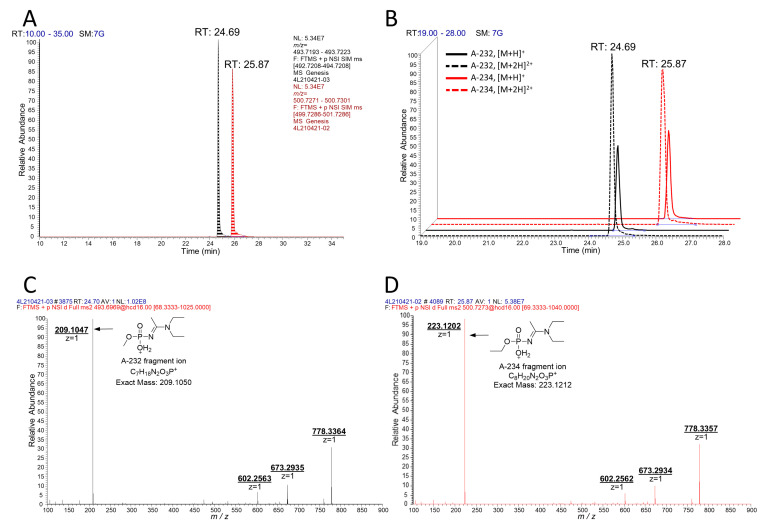
LC-MS/MS analysis of [M+2H]^2+^ ions of A232- or A234-inhibited BChE with pepsin digestion. (**A**) Extracted chromatograms from tSIM of biomarkers from A232 (black) and A234 (red), and their abundances compared with [M+H]^+^ ions (**B**–**D**) are the MS2 spectra of the biomarkers from A232 and A234, respectively.

**Figure 6:**
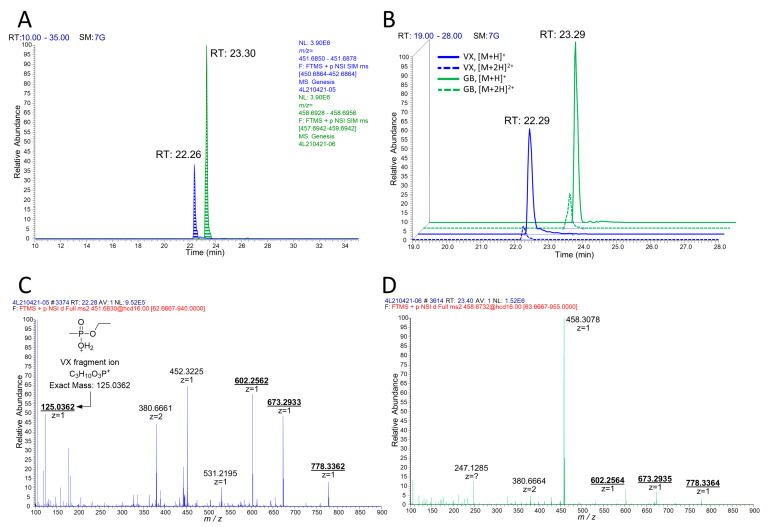
LC-MS/MS analysis of [M+2H]^2+^ ions of VX- or GB-inhibited BChE with pepsin digestion. (**A**) Extracted chromatograms from tSIM of biomarkers from VX (blue) and GB (green), and their abundances are compared with [M+H]^+^ ions (**B**–**D**) are the MS2 spectra of the biomarkers from VX and GB, respectively.

When analyzing peptides with LC-API-MS/MS, the richness of basic sites on typical peptides makes multiply-charged molecular ions much more dominant than singly charged molecular ions ([M+H]^+^ ions) [24]. However, studies preparing and detecting biomarkers for organophosphate-inhibited BChE have commonly used [M+H]^+^ ions [18,19,20,21,22,23]. Since the nonapeptide obtained from BChE by pepsin digestion does not contain basic residues, such as arginine, histidine or lysine, the generation of multiply charged molecular ions does not seem plausible in this case. We attempted to determine whether the biomarker with the Novichok agent might share the same tendency on ionization by identifying [M+2H]^2+^ ions from the prepared sample (Figure 5). Interestingly, the abundance of [M+2H]^2+^ ions of the biomarkers was quite higher than that of [M+H]^+^ ions, in contrast to the VX- and GB-inhibited biomarkers prepared and analyzed in the same way (Figure 6). These [M+2H]^2+^ ions were much weaker against fragmentation, resulting in a 30% lower optimized collision energy. The fragment ion obtained directly from the inhibiting Novichok agent became a base peak of [M+2H]^2+^ ions on MS2 fragmentation, since its intensity was more than 10 times higher than that of the [M+H]^+^ ion analysis. This characteristic also made [M+2H]^2+^ more suitable for unique verification.

We also detected [M+2H]^2+^ ions for VX- and GB-inhibited biomarkers, but their abundances were much lower, and the nerve agent fragment ions did not form a base peak. Interestingly, there was additional difference between MS2 spectra of GB and VX. There was a fragment ion peak at VX while GB did not show any relevant peak. This might be the result of reduced collision energy. Overall intensity of peptide ions are much more suppressed in GB than VX—while the MS1 intensity of GB is 2.5 times bigger. The peak at *m*/*z* 452.3225 in VX adduct analysis and *m*/*z* 458.3078 in GB adduct analysis came from the pepsin-digested sample itself, which was coincidently injected through the quadrupole (*m*/*z* filter width 2.0).

### 2.3. Comparing [M+H]^+^ and [M+2H]^2+^ Ion Ratio between Nerve Agents

The analyzed ion ratios of [M+H]^+^ and [M+2H]^2+^ ions of each nerve agent was calculated and compared (Table 1). Novichok nerve agents showed clearly different trends from the ratios obtained for VX and GB, and it was clear that detecting [M+2H]^2+^ ions would increase the sensitivity of analysis.

## 3. Discussion

Although we discovered extraordinary increase in intensity of [M+2H]^2+^ ions for Novichok-inhibited biomarkers, we have not found the exact reason for difference in ionization. Changing ionization source parameters did not affect this phenomenon (data can be found in Supplementary Materials). We believe that the structure of nerve agents might play a role, compared to the data from VX and GB, so we are going to find a relation between the structure and ion ratio by researching with other OPNAs.

The nerve agent fragment ion was another distinguishing characteristic of Novichok agents. This would make identifying the species of nerve agent easier, as this observation is unique for Novichok-inhibited BChE. For this finding, we postulate that the high resistance of Novichok agents against hydrolysis, studied in our previous research, could be an answer. The presence of the VX fragment ion in [M+2H]^2+^ ion analysis, not in [M+H]^+^ ion due to higher collision energy supports the postulation. Nevertheless, the lack of previous research keep us from concluding. We will collect more information to confirm the relation between the stability and this characteristic.

## 4. Materials and Methods

### 4.1. Synthesis of the Chemicals

All chemicals and reagents required for the microsynthesis of A232 (CAS 2387496-04-8), A234 (CAS 2387496-06-0), VX, and GB were purchased from Sigma-Aldrich (Seoul, Korea). Solvents for LC-MS/MS were purchased from Merck (Seoul, Korea). All agents were micro-synthesized in our laboratory, and the synthesis result was verified by nuclear magnetic resonance spectroscopy (^1^H and ^31^P NMR spectra of synthesized agents are in Supplementary Information). The purity of the agents was over 95%.

### 4.2. Nerve-Agent Inhibited Human BChE Preparation

Human BChE adducts for A232 and A234 were produced using EDTA-inhibited pooled human serum (Innovative Research, Novi, MI, USA). Serum samples were spiked with 20 µg/mL of each nerve agent (A232, A234, VX, and GB) and incubated in a shaking incubator at 37 °C for 16 h. Spiked serum samples were mixed with anti-BChE antibody (HAH-002-01-02, Thermo Fisher Scientific, Seoul, Korea) conjugated with magnetic beads (Cat. No. 14204, Thermo Fisher Scientific, Seoul, Korea) and then gently agitated at room temperature for 1 h. Conjugation of the beads with anti-BChE antibody was performed following the kit’s instructions. The supernatant of the mixture was separated and discarded using a magnet, and the beads were washed twice with phosphate-buffered saline (PBS). Then, the cells were treated with 400 µg/mL pepsin (Thermo Fisher Scientific, Seoul, Korea) in 10 mM acetic acid and incubated at 37 °C for 3 h with shaking. After incubation, the sample was filtered with a 10 kDa MWCO filter, and the filtrate was analyzed with LC-MS/MS.

### 4.3. RSLC Conditions

An Ultimate 3000 RSLC system equipped with a Thermo Fisher PepMap C18 column (150 mm × 150 µm, 2 µm particle size) was used for the analysis. The mobile phase consisted of water (Solvent A) and acetonitrile (Solvent B) with 0.1% formic acid. Samples (1 µL) were initially trapped with a PM100 C18 column (20 mm × 75 µm, 3 µm particle size) with loading buffer (98% water, 2% acetonitrile with 0.05% formic acid) at 3 µL/min for 5 min and then eluted with RSLC flow. The gradient conditions were as follows: 12% B from 0 to 10 min, linear increase to 40% B at 29 min, increase to 95% B at 30 min, hold for 5 min, decrease to 12% B at 35 min, and hold for 5 min. The flow rate was set to 300 nL/min.

### 4.4. Mass Spectrometer Conditions

The effluent was transferred to an Orbitrap Q Exactive (Thermo Fisher Scientific, San Jose, CA, USA) with an atmospheric pressure ionization source/interface operated in nanoscale electrospray ionization (nano-ESI) mode using an Easy-Spray Source. The capillary temperature and spray voltage were optimized to obtain a maximum response in the *m*/*z* range from 500–1000. Data for optimizations can be found in Supplementary Information. The nano-ESI conditions were as follows: spray voltage 1.7 kV and capillary temperature 270 °C. Nitrogen was used for the collision gas at 120 psi. Xcalibur software (Thermo Fisher, San Jose, CA, USA) was used as a control instrument to acquire and process the data.

## 5. Conclusions

In this research, we searched for biomarkers in human plasma for the Novichok nerve agents A232 and A234, derived from organophosphate-inhibited BChE. These new agents formed similar biomarkers with BChE as the conventional warfare agents do, by covalently inhibiting same active site residue. This result supports our assumption that they might intoxicate human same as their ancestral relatives.

However, biomarkers from Novichok-inhibited BChE were different from biomarkers from VX or GB. First, the abundance of [M+2H]^2+^ ions is much higher than [M+H]^+^ ions in Novichok-inhibited biomarkers. Second, characteristic nerve agent fragment ions appear in MS2 fragmentation. The relative intensities between peptide fragment ions in MS2 spectra also exhibited difference. Biomarkers from Novichok have higher intensity for higher *m*/*z* fragments, just opposite to the biomarkers from VX and GB. Finally, [M+2H]^2+^ ions are better for trace analysis of biomarkers owing to higher intensities and unique MS2 fragment ions, they also have problems with suppressed intensities of peptide fragment ions, which is also useful for peptide identification. For this reason, it is suggested to use both molecular ions in trace analysis for richer information.

## Figures and Tables

**Table 1:** Major fragment ions from A232 or A234-inhibited butyrylcholinesterase.

Agent	Peak Area * of [M+H]^+^ Ion	Peak Area * of [M+2H]^2+^ Ion	Area Ratio ([M+H]^+^/[M+2H]^2+^)
A232	3.01 × 10^8^	8.29 × 10^8^	0.363 ± 0.074
A234	3.05 × 10^8^	4.74 × 10^8^	0.644 ± 0.121
VX	2.59 × 10^8^	2.93 × 10^7^	8.984 ± 1.750
GB	2.00 × 10^8^	3.33 × 10^7^	6.011 ± 0.791

* Peak area is averaged from three independent analyses.

## Data Availability

Data is contained within Supplementary Materials.

## Supplementary Materials

The following are available online, Figures S1–S4: ^1^H NMR spectra and ^31^P NMR spectra of synthesized A232/A234/VX/GB, Figures S5–S8: [M+Na]^+^ and [M+H+Na]^2+^ ion observations for A232/A234/VX/GB-inhibited BChE with pepsin digestion for proving major ion species, Figure S9 to S10: Source optimization (capillary temperature) for analysis of A234/VX-inhibited BChE with pepsin digestion, Figures S11–S12: Source optimization (spray voltage) for analysis of A234/VX-inhibited BChE with pepsin digestion.

## Sample Availability

All synthesized compounds in this research are not available for distribution—they are regulated under Chemical Weapons Convention.
