# Supplementary material for: Identification and Study of Biomarkers from Novichok-Inhibited Butyrylcholinesterase in Human Plasma
Source: Molecules. 2021 Jun 22;26(13):3810. doi: 10.3390/molecules26133810 (PMC8270327; doi:10.3390/molecules26133810)
Supplement: Supplementary file 1 [file molecules-26-03810-s001.zip › molecules-1254731-supplementary-final.pdf]

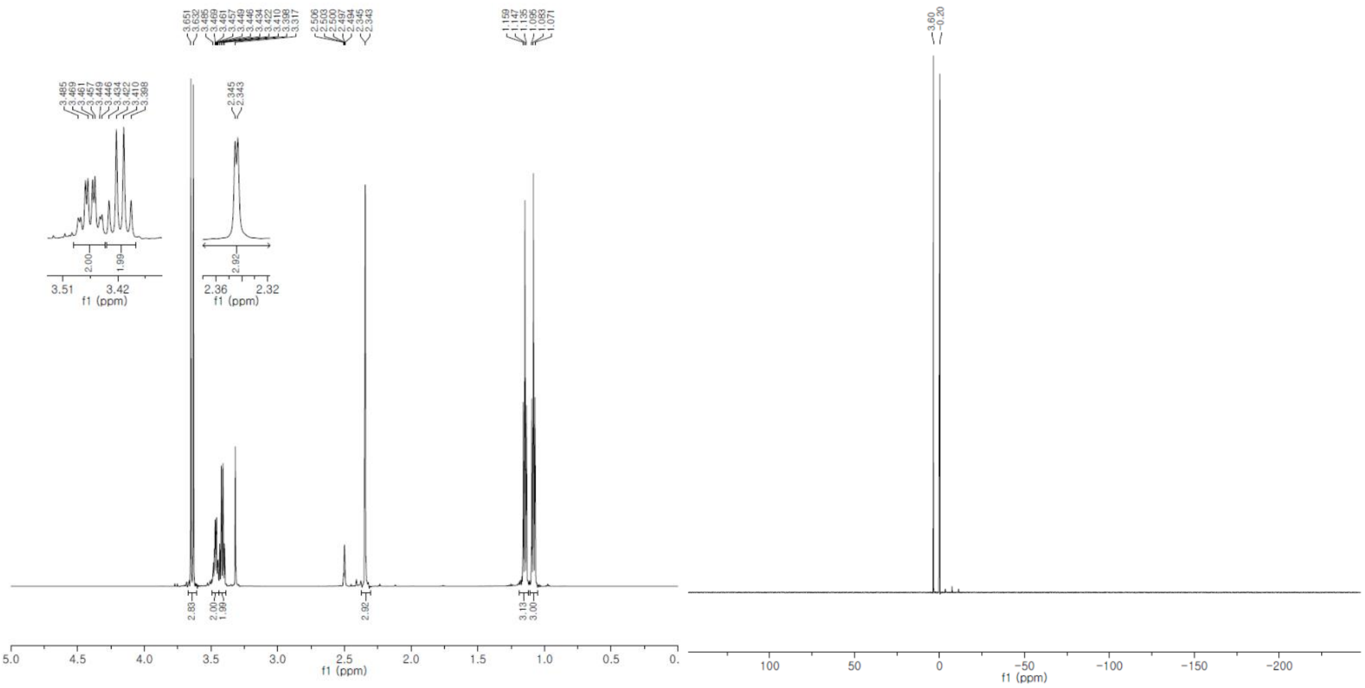

Supplementary Figure S1. ^{1}H NMR spectra (left) and ^{31}P NMR spectra (right) of synthesized A232.

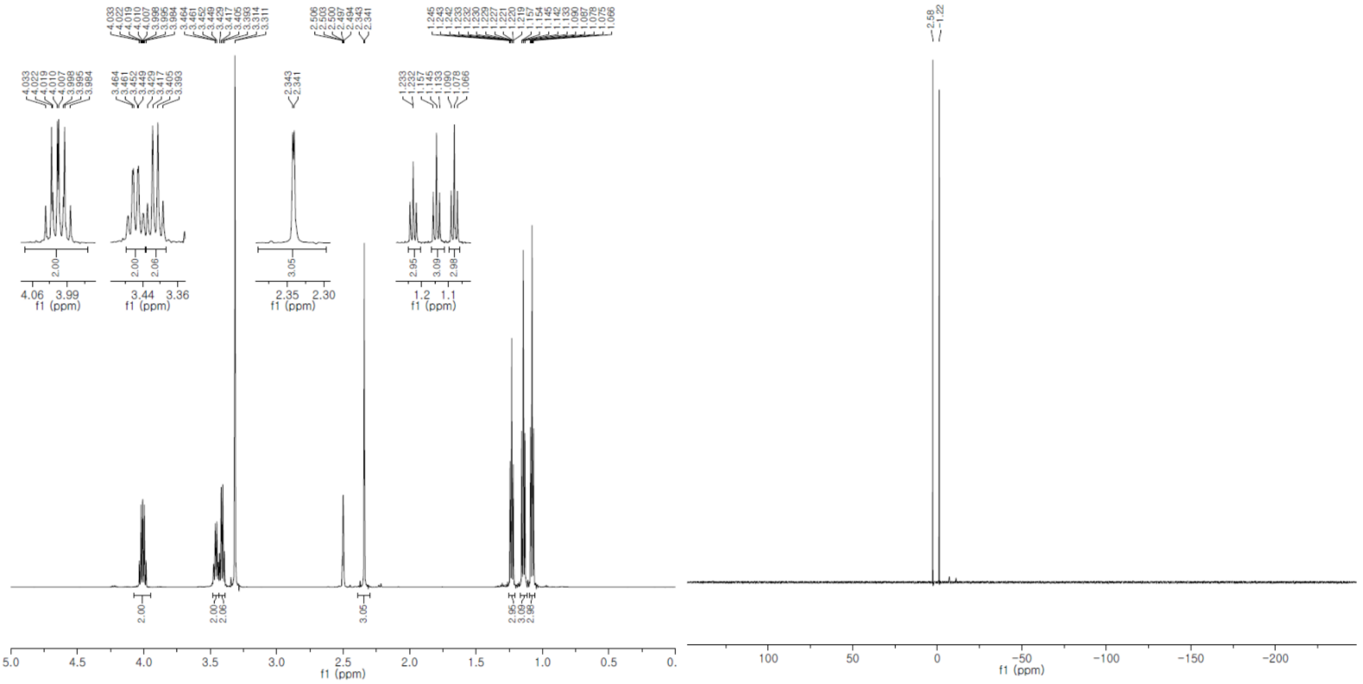

Supplementary Figure S2. ^{1}H NMR spectra (left) and ^{31}P NMR spectra (right) of synthesized A234.

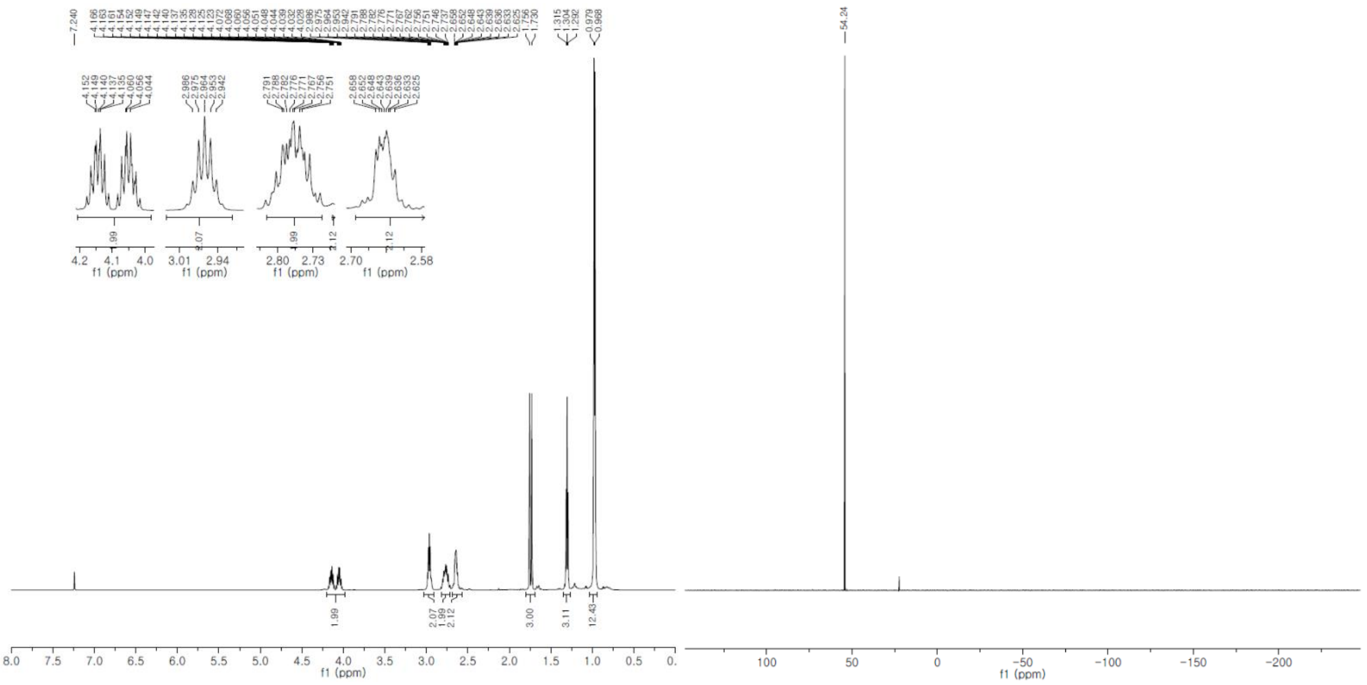

Supplementary Figure S3. ^{1}H NMR spectra (left) and ^{31}P NMR spectra (right) of synthesized VX.

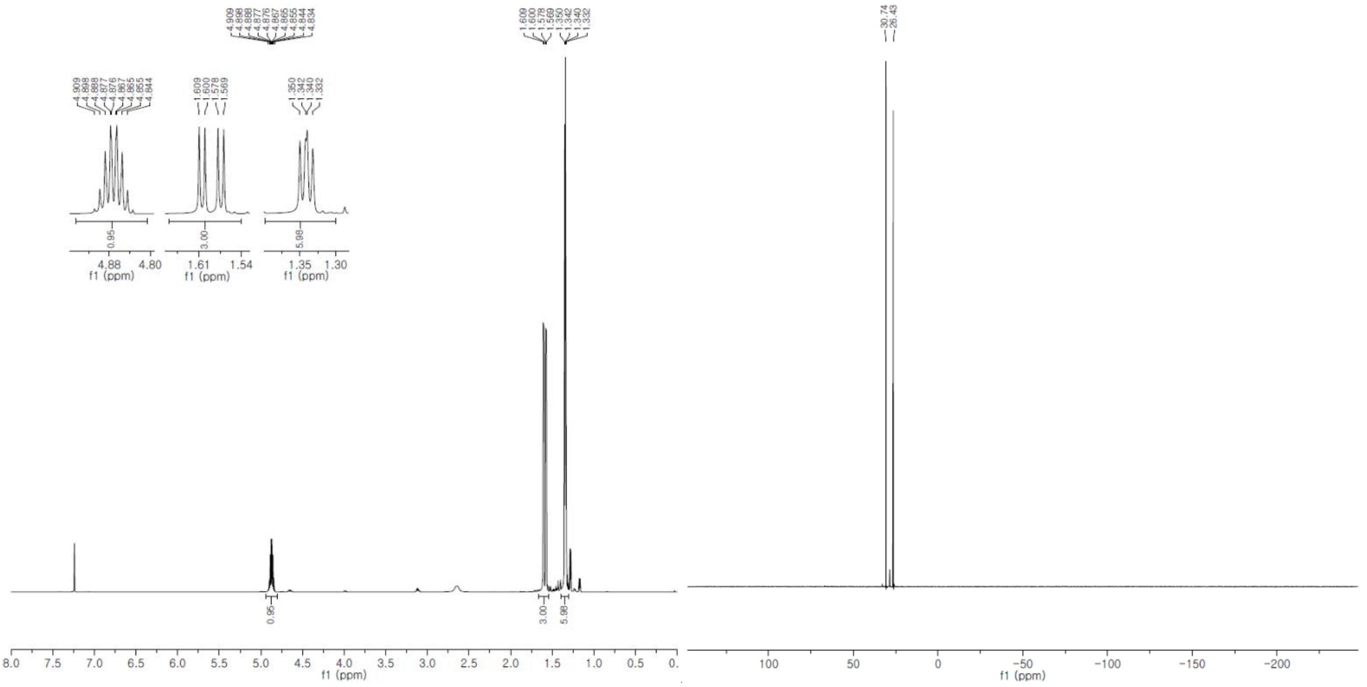

Supplementary Figure S4. ^{1}H NMR spectra (left) and ^{31}P NMR spectra (right) of synthesized GB.

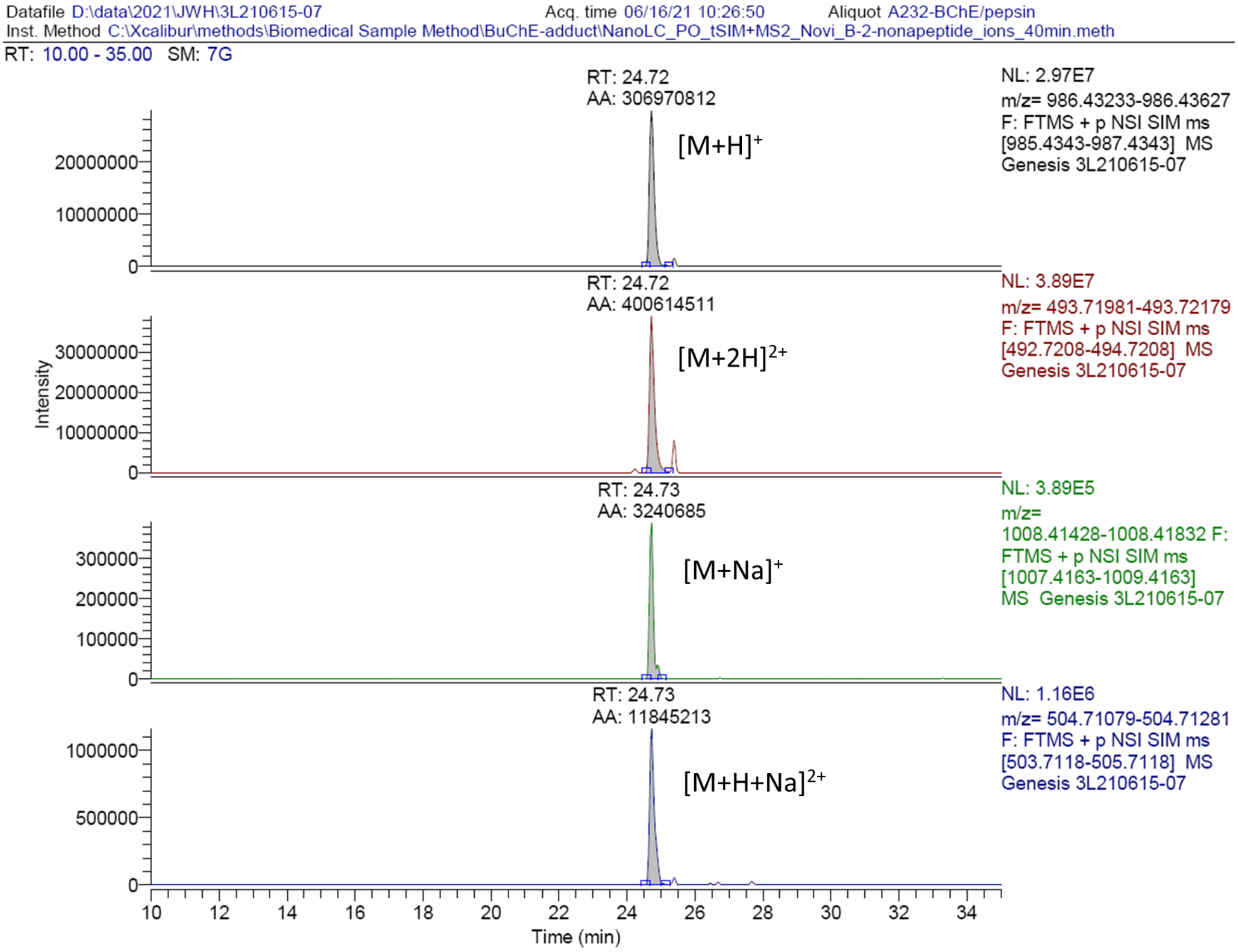

Supplementary Figure S5. $[M+Na]^+$ and $[M+H+Na]^{2+}$ ion observation for A232-inhibited BChE with pepsin digestion to prove that generation of those ions did not affect the intensity of $[M+H]^+$ or $[M+2H]^{2+}$ ions.

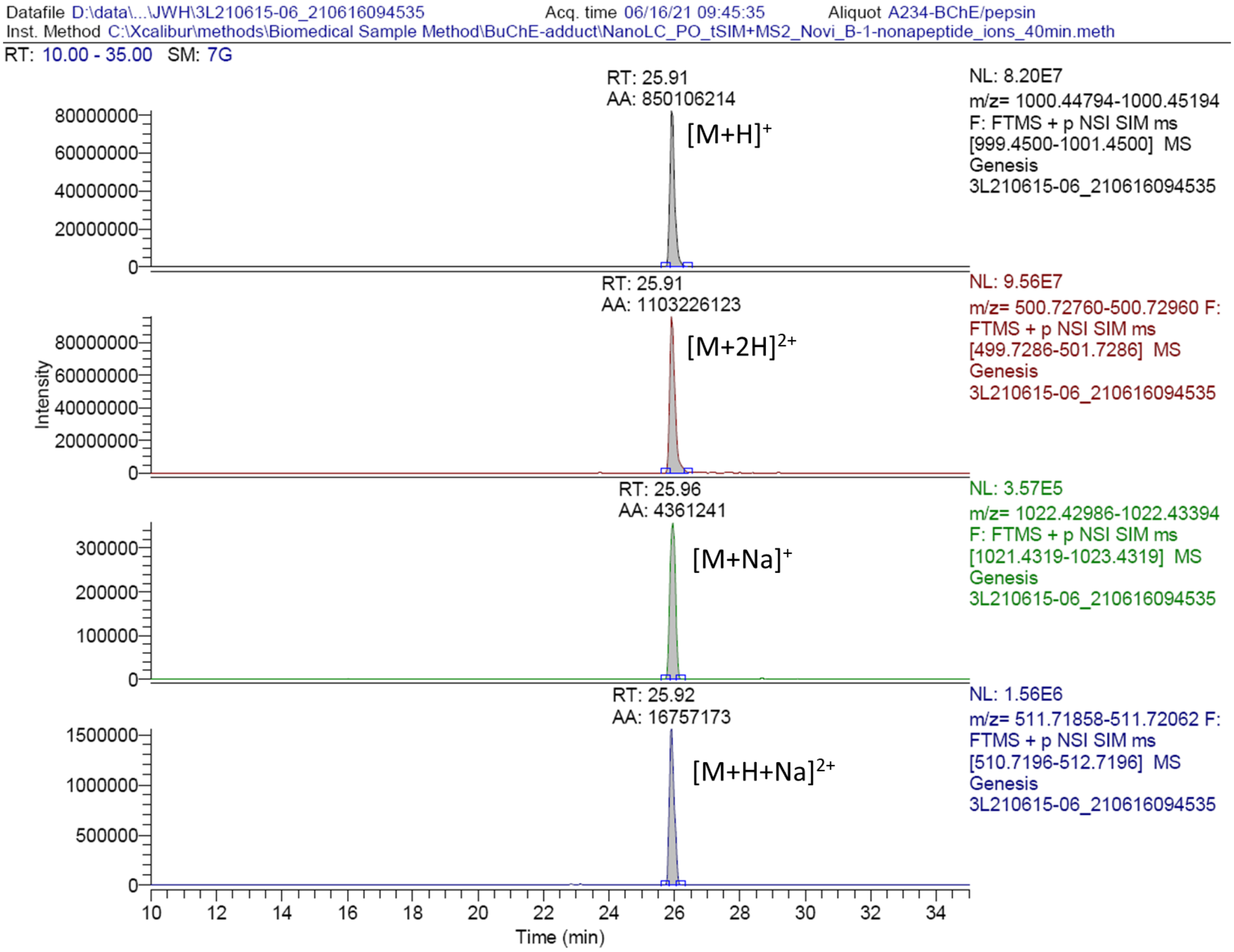

Supplementary Figure S6. $[M+Na]^+$ and $[M+H+Na]^{2+}$ ion observation for A234-inhibited BChE with pepsin digestion to prove that generation of those ions did not affect the intensity of $[M+H]^+$ or $[M+2H]^{2+}$ ions.

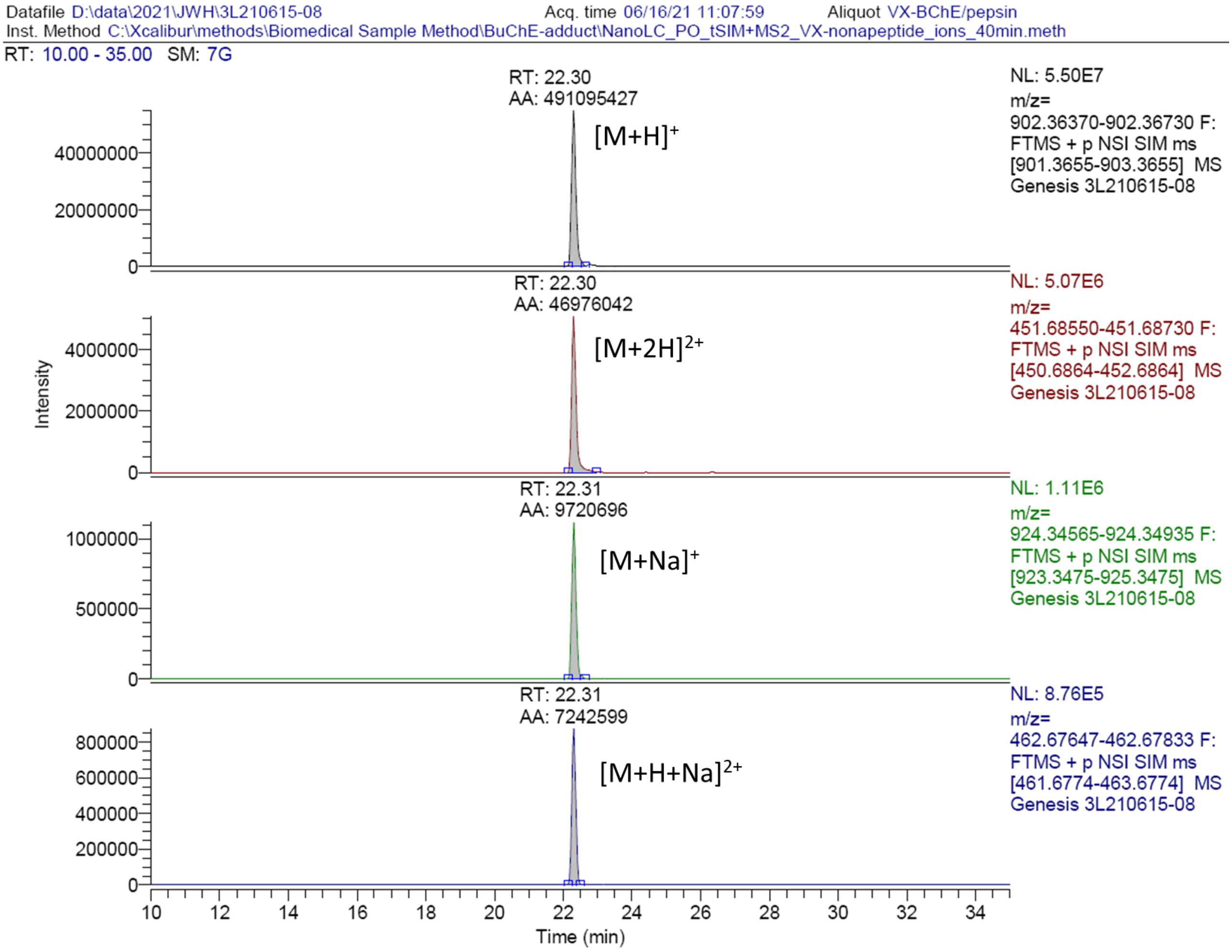

Supplementary Figure S7. $[M+Na]^+$ and $[M+H+Na]^{2+}$ ion observation for VX-inhibited BChE with pepsin digestion to prove that generation of those ions did not affect the intensity of $[M+H]^+$ or $[M+2H]^{2+}$ ions.

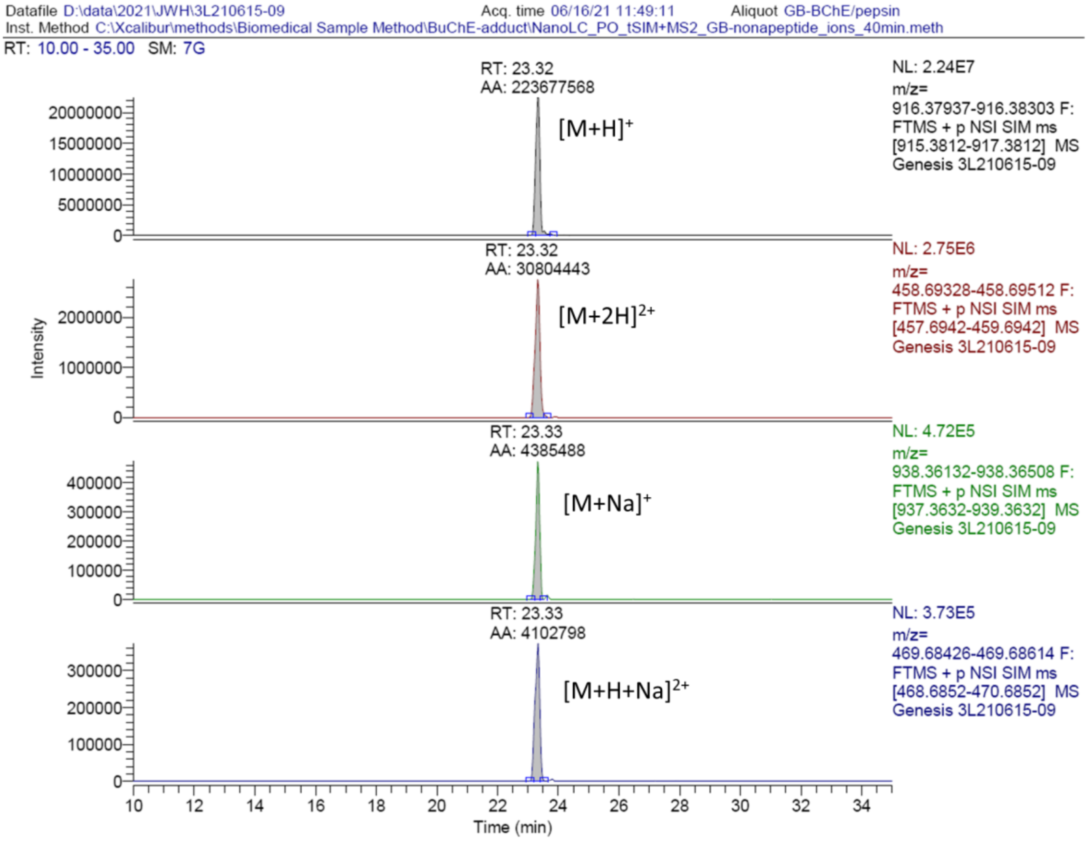

Supplementary Figure S8. $[M+Na]^+$ and $[M+H+Na]^{2+}$ ion observation for GB-inhibited BChE with pepsin digestion to prove that generation of those ions did not affect the intensity of $[M+H]^+$ or $[M+2H]^{2+}$ ions.

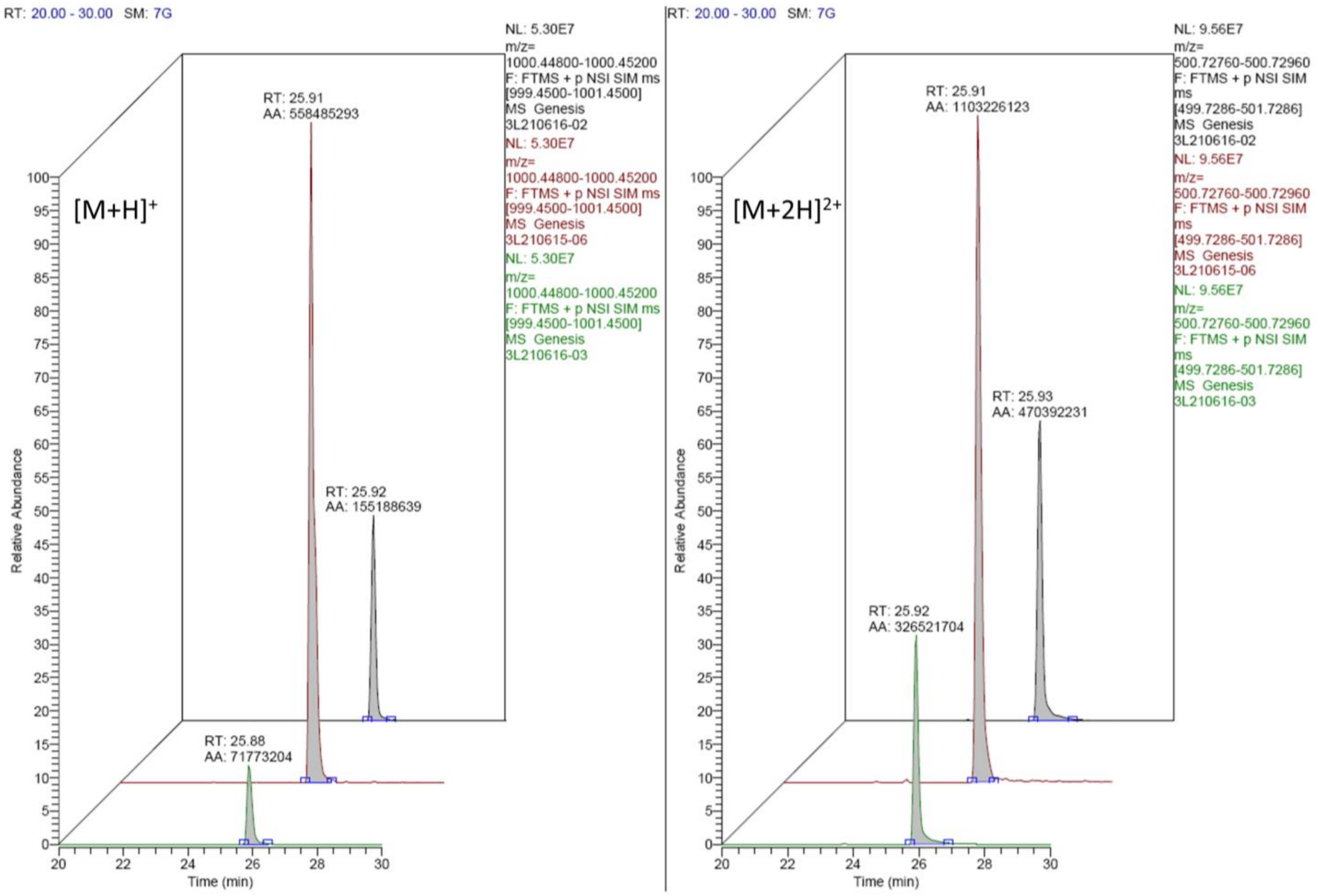

Supplementary Figure S9. Source optimization for analysis of A234-inhibited BChE with pepsin digestion. Capillary temperature was tested for 240 (black), 270 (red), and 300 degrees (green) and $[M+H]^+$ (left) and $[M+2H]^{2+}$ (right) were monitored simultaneously.

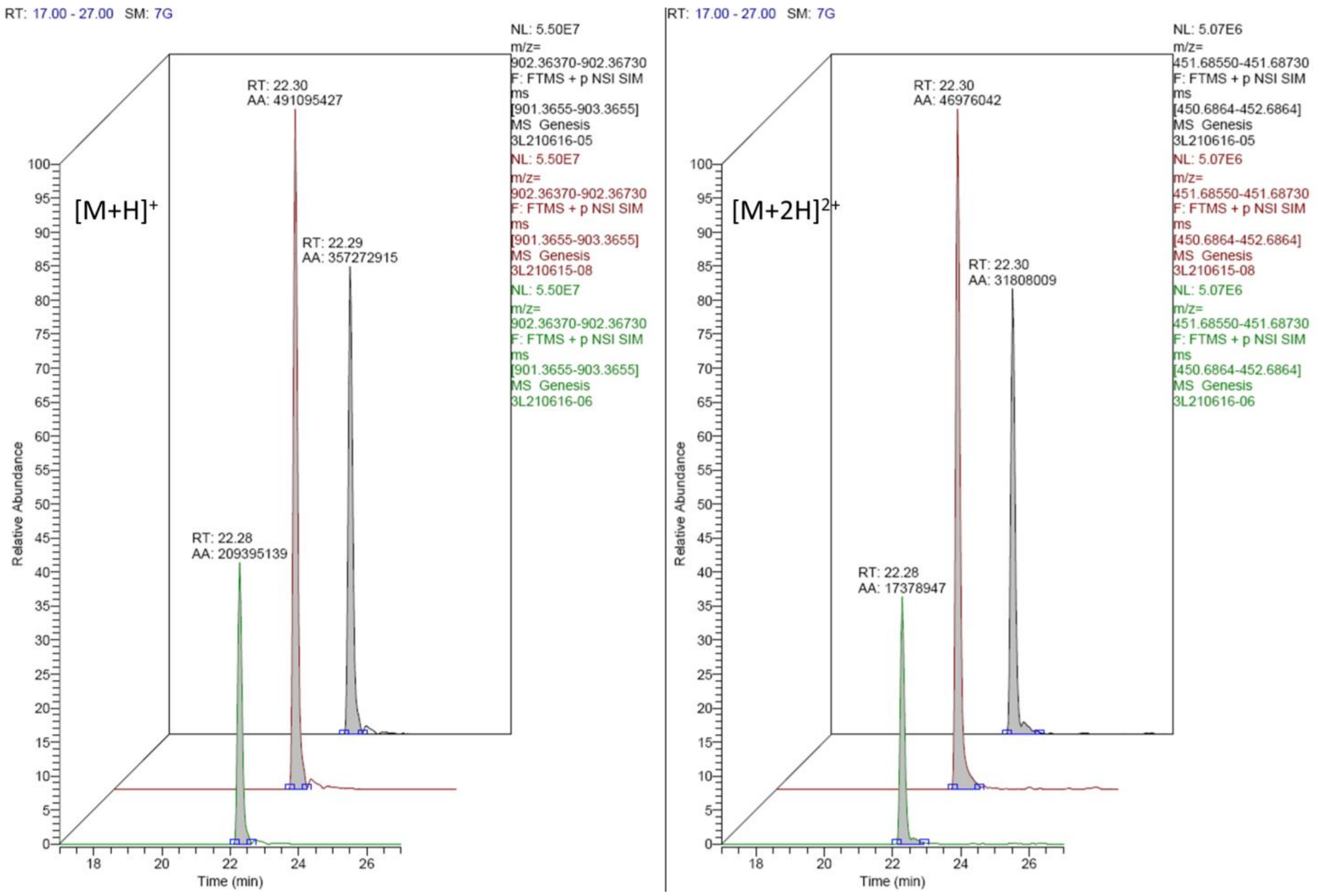

Supplementary Figure S10. Source optimization for analysis of VX-inhibited BChE with pepsin digestion. Capillary temperature was tested for 240 (black), 270 (red), and 300 degrees (green) and $[M+H]^+$ (left) and $[M+2H]^{2+}$ (right) ions were monitored simultaneously.

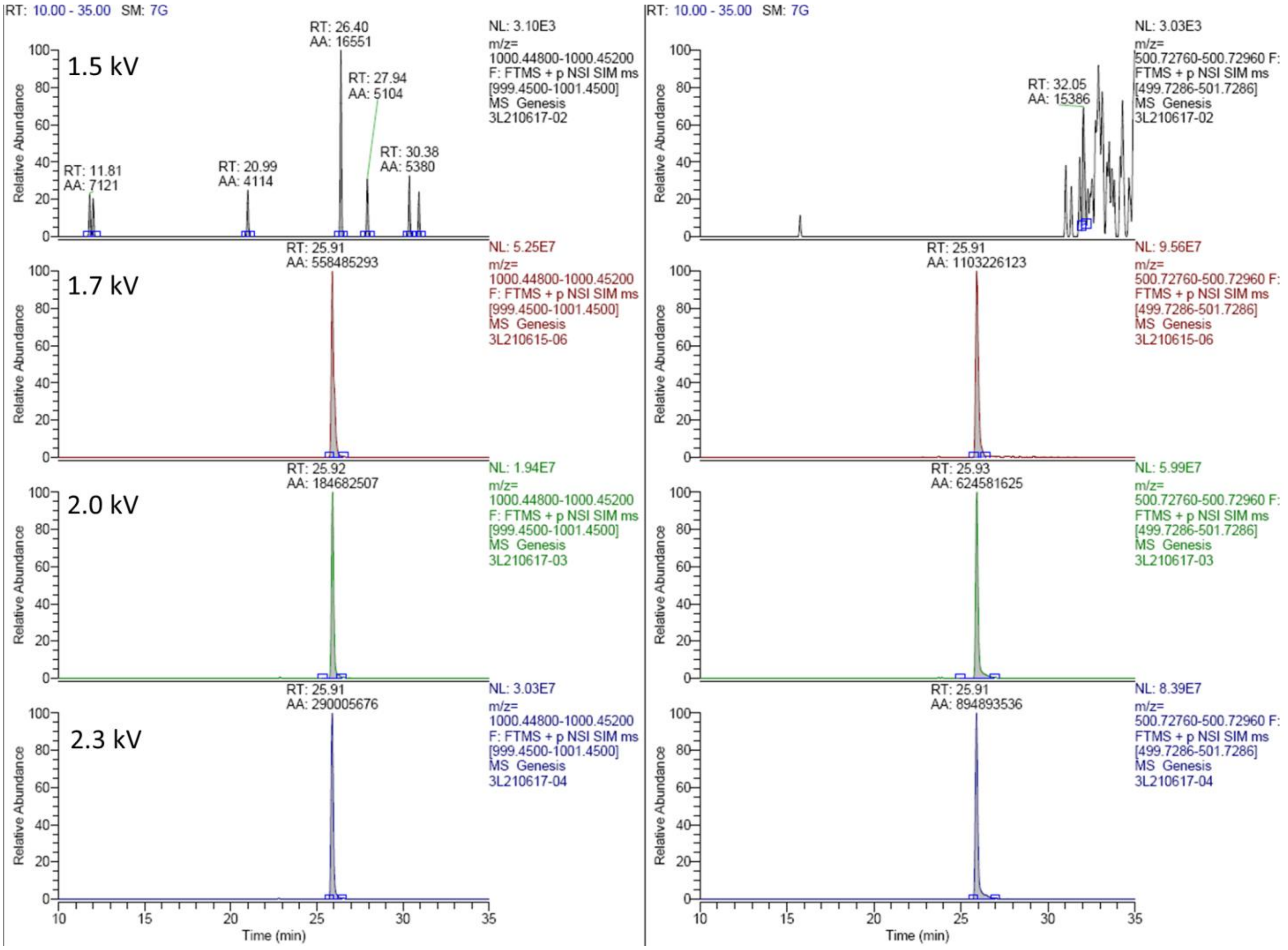

Supplementary Figure S11. Source optimization for analysis of A234-inhibited BChE with pepsin digestion. Spray voltage was tested for 1.5, 1.7, 2.0 and 2.3 kV as annotated in the figure. $[M+H]^+$ (left) and $[M+2H]^{2+}$ (right) ions were monitored simultaneously.

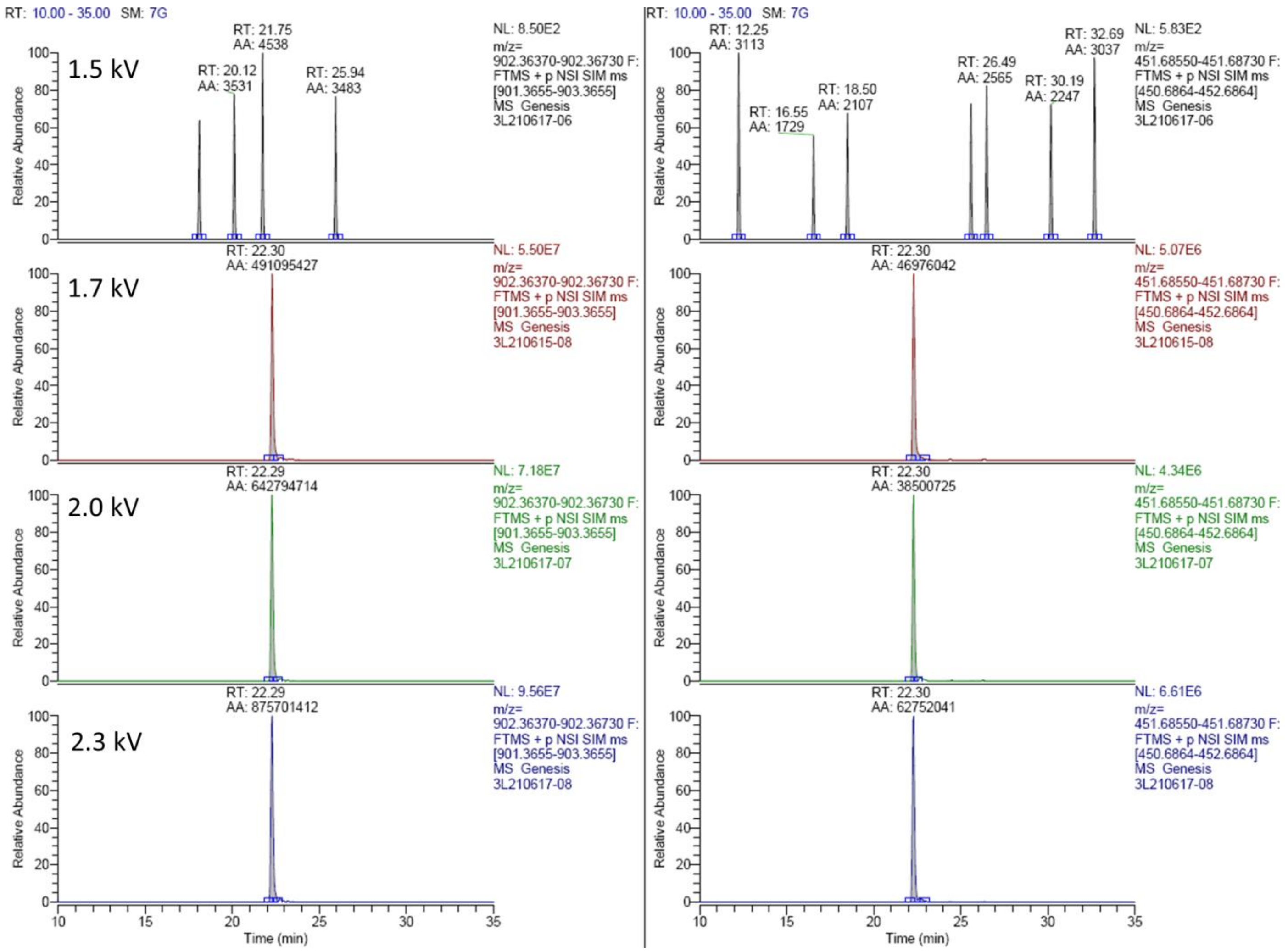

Supplementary Figure S12. Source optimization for analysis of VX-inhibited BChE with pepsin digestion. Spray voltage was tested for 1.5, 1.7, 2.0 and 2.3 kV as annotated in the figure. $[M+H]^+$ (left) and $[M+2H]^{2+}$ (right) ions were monitored simultaneously.
